# WAPL induces cervical intraepithelial neoplasia modulated with estrogen signaling without HPV E6/E7

**DOI:** 10.1038/s41388-021-01787-5

**Published:** 2021-05-04

**Authors:** Katsuyoshi Kumagai, Masakatsu Takanashi, Shin-ichiro Ohno, Yuichirou Harada, Koji Fujita, Keiki Oikawa, Katsuko Sudo, Shun-ichi Ikeda, Hirotaka Nishi, Kosuke Oikawa, Masahiko Kuroda

**Affiliations:** 1 Pre-clinical Research Center, Tokyo Medical University, Tokyo, Japan; 2 Department of Molecular Pathology, Tokyo Medical University, Tokyo, Japan; 3 Department of Obstetrics and Gynecology, Kohseichuo General Hospital, Tokyo, Japan; 4 Department of Obstetrics and Gynecology, Tokyo Medical University, Tokyo, Japan; 5 Department of Pathology, Wakayama Medical University, Wakayama, Japan

**Keywords:** Cervical cancer, Hormone receptors

## Abstract

Since cervical cancer still afflicts women around the world, it is necessary to understand the underlying mechanism of cervical cancer development. Infection with HPV is essential for the development of cervical intraepithelial neoplasia (CIN). In addition, estrogen receptor signaling is implicated in the development of cervical cancer. Previously, we have isolated human wings apart-like (WAPL), which is expected to cause chromosomal instability in the process of HPV-infected precancerous lesions to cervical cancer. However, the role of WAPL in the development of CIN is still unknown. In this study, in order to elucidate the role of WAPL in the early lesion, we established WAPL overexpressing mice (WAPL Tg mice) and HPV E6/E7 knock-in (KI) mice. WAPL Tg mice developed CIN lesion without HPV E6/E7. Interestingly, in WAPL Tg mice estrogen receptor 1 (ESR1) showed reduction as compared with the wild type, but cell growth factors MYC and Cyclin D1 controlled by ESR1 expressed at high levels. These results suggested that WAPL facilitates sensitivity of ESR1 mediated by some kind of molecule, and as a result, affects the expression of MYC and Cyclin D1 in cervical cancer cells. To detect such molecules, we performed microarray analysis of the uterine cervix in WAPL Tg mice, and focused MACROD1, a co-activator of ESR1. MACROD1 expression was increased in WAPL Tg mice compared with the wild type. In addition, knockdown of WAPL induced the downregulation of MACROD1, MYC, and Cyclin D1 but not ESR1 expression. Furthermore, ESR1 sensitivity assay showed lower activity in WAPL or MACROD1 downregulated cells than control cells. These data suggested that WAPL increases ESR1 sensitivity by activating MACROD1, and induces the expression of MYC and Cyclin D1. Therefore, we concluded that WAPL not only induces chromosomal instability in cervical cancer tumorigenesis, but also plays a key role in activating estrogen receptor signaling in early tumorigenesis.

## Introduction

Persistent HPV infection causes cervical squamous epithelial cells to transform into cervical intraepithelial neoplasia (CIN) [1, 2]. In particular, HPV E6/E7 oncoproteins induce immortality and multistep carcinogenesis of cervical squamous epithelial cells [3, 4]. We have previously reported that HPV E6/E7 increases wings apart-like (WAPL) expression, and WAPL activation in the lesions of CIN and advanced cervical cancer is involved in tumorigenesis and tumor progression [5, 6]. On the other hand, WAPL is involved in the regulation of the interaction between cohesin and chromatin. WAPL destabilizes cohesin binding to chromatin, but is inhibited in early mitosis by Sororin to maintain stable sister chromatid cohesion. Thus, WAPL is an essential factor for chromatin structure and chromosome segregation [7–12]. Furthermore, we have previously shown that high-level expression of WAPL is involved in chromosomal instability [13]. However, it is still unknown whether WAPL plays a direct role in the induction of CIN, which is an early lesion of cervical cancer. Estrogen contributes to the development of cancers such as breast, ovarian, and colorectal cancer by estrogen receptor (ER)-dependent and independent mechanisms. In HPV-associated cancer model mouse, estrogen and its receptor estrogen receptor 1 (ESR1) are considered to be essential for the development of cervical cancer [14–16]. ESR1 plays a critical role at the early stage of cervical carcinogenesis [16–19]. However, cervical cancer-derived cell lines and later stage cervical cancer show low-level expression of ESR1 [20, 21]. Furthermore, ESR1 is unnecessary in the process of the progression from high-grade squamous intraepithelial lesions (HSIL)/CIN3 to squamous cell carcinoma (SCC) [20–23]. Therefore, the role of ESR1 in the development of CIN and the significance of ESR1 reduction in CIN3 and SCC are not sufficiently clear. MACROD1 (Mono-ADP-ribosylhydrolase 1) is a macrodomain containing protein that has mono-ADP-ribose hydrolase enzymatic activity, which is also known as leukemia-related protein 16 (LRP16). MACROD1 is a co-activator of ESR1 and upregulates ESR1 target genes including MYC and Cyclin D1 [24]. In addition, knockdown (KD) of MACROD1 in estrogen-resistant ovarian cancer cells markedly reduces estrogen response element-dependent ESR1 reporter gene activity and estrogen-induced MYC expression [25]. However, there have been no reports of an association between MACROD1 and cervical cancer. In this study, we found that WAPL overexpressing mice developed CIN without HPV E6/E7. MACROD1 was enhanced in the uterine cervix of the WAPL overexpressing mice. We also found that KD of WAPL in human cervical cancer cells reduced estrogen sensitivity and expression of ESR1 downstream genes, MYC and Cyclin D1. These data indicated that WAPL associates with MACROD1, influencing the estrogen sensitivity of ESR1 and the expression of MYC and Cyclin D1. Thus, we conclude that WAPL plays a key role in activating estrogen receptor signaling in early tumorigenesis of CIN without involvement of HPV E6/E7.

## Results

### Development of CIN in the cervical squamous epithelium by the high-level expression of WAPL without HPV E6/E7

HPV E6/E7 transgenic (Tg) mice treated with estrogen develop CIN [14]. On the other hand, our previous data have suggested that HPV E6/E7 induce WAPL expression, and overexpression of WAPL in CIN is involved in cervical tumorigenesis and tumor progression [5, 6]. From these results, we considered that WAPL may be implicated in the development of CIN. In this study, to clarify the mechanism of WAPL-mediated cervical carcinogenesis, we examined whether the development of CIN was caused directly by WAPL overexpression instead of the effect of HPV E6/E7. On this account, we generated WAPL transgenic (Tg) mice and HPV E6/E7 knock-in (KI) mice (Supplementary Fig.1).

**Fig. 1:**
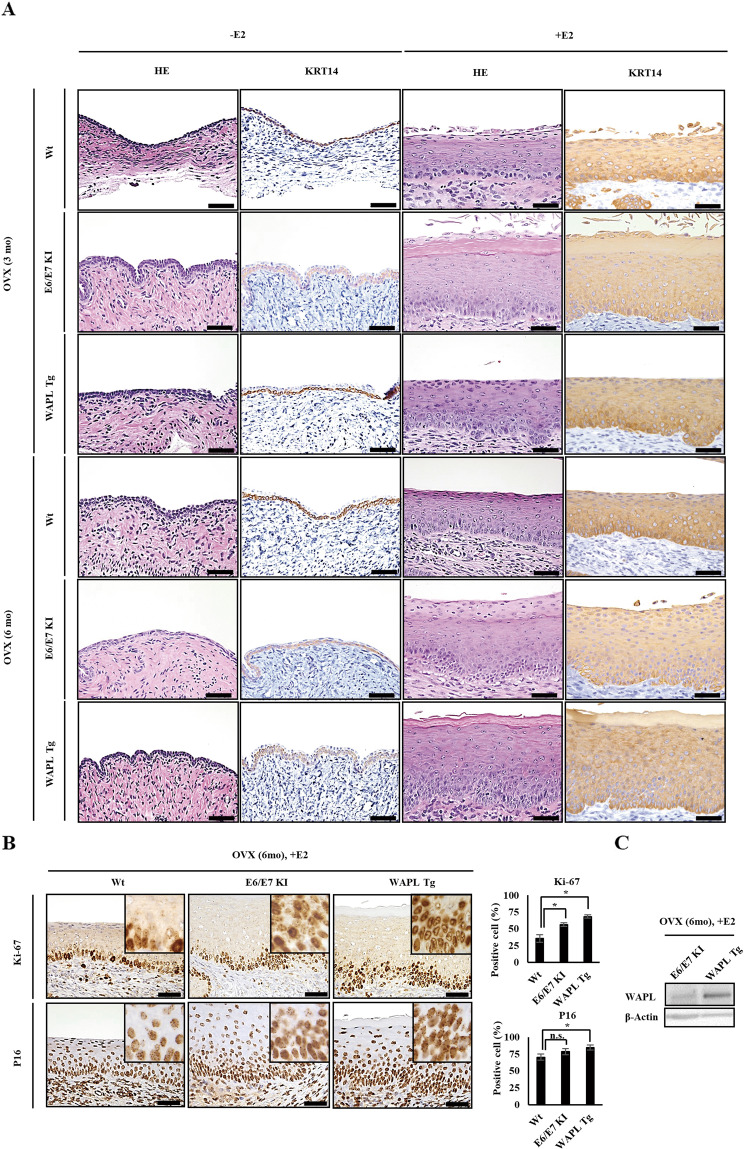
CIN of the cervical squamous epithelium in OVX-treated WAPL Tg mice injected with E2. **A** HE stain and IHC analysis for Keratin 14 (KRT14) in cervical squamous epithelium of Wt, HPV E6/E7 KI and WAPL Tg mice at 3 and 6 months after OVX. The mice were divided into two groups, the E2-injected group and non-E2-injected group. The scale bars represent 50 μm. **B** Expression of Ki-67 and P16 in cervical squamous epithelium of E2-injected Wt, HPV E6/E7 KI and WAPL Tg mice at 6 months after OVX. Left panels show IHC analysis for Ki-67 and P16. Insets show higher magnification images. The scale bars represent 50 μm. Right panels show the graphs representing the rates of Ki-67 and P16 positive cells in the cervical squamous epithelium. **C** Comparison of WAPL protein expression in the uterine cervix between E2-injected HPV E6/E7 KI and WAPL Tg mice at 6 months after OVX by western blot analysis. The data were obtained from 6 mice for each group, and the representative images are shown. Error bars on the graphs in **B** represent SD of the mean. Statistical significance was calculated by Student *t* test. *p < 0.05. n.s. no significant difference.

In rodents, ovarian steroid hormones cause morphological changes in the fallopian tubes, uterus, and upper vagina [26–29]. In order to analyze the association between estrogen and WAPL, WAPL Tg mice need to be suppressed endogenous estrogen secretion and given a certain amount of estrogen. Therefore, we first performed ovariectomy (OVX) to wild type (Wt), HPV E6/E7 KI, and WAPL Tg mice. Then, we examined whether the OVX mice injected with 17β-Estradiol (E2), the predominant form of estrogen, developed CIN. We failed to find CIN of cervical squamous epithelium in a non-E2-injected group of the mice at 3 and 6 months after OVX (Fig. 1A). In the E2-injected group, however, although abnormalities in the cervical squamous epithelium were not observed in the WAPL Tg mice at 3 months after OVX, the WAPL Tg mice at 6 months after OVX showed dysplastic change of the cervical squamous epithelium (Fig. 1A). Because Ki-67 and P16 are indicators of HSIL/CIN3 [30, 31], screening using a combination of Ki-67 and P16 is expected to be useful for detection of CIN and SCC [32, 33]. Therefore, we performed immunohistochemistry (IHC) analysis using Ki-67 and P16 on the cervical squamous epithelium of the mice. The results showed that the rate of the Ki-67 and P16 positive cells in cervical squamous epithelium of the E2-injected WAPL Tg mice at 6 months after OVX was higher than that of the Wt mice (Fig. 1B). Furthermore, the phenotype of the OVX-WAPL Tg mice was severe compared to the E6/E7 KI mice (Fig. 1A). Western blot analysis confirmed that the WAPL expression level in the uterine cervix of the WAPL Tg mice was higher than that of the E6/E7 KI mice (Fig. 1C). These results suggested that the high-level expression of WAPL in the cervical squamous epithelium developed CIN under the effect of estrogen without HPV E6/E7.

### Inconsistent expression of ESR1 and its downstream molecules in OVX-WAPL Tg mice

**Fig. 2:**
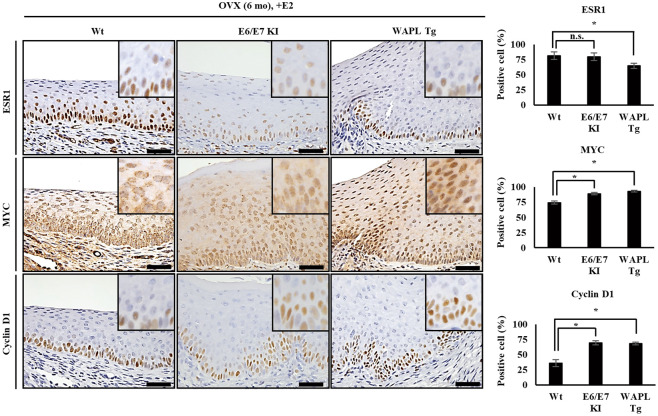
Comparison of the expression of ESR1, MYC, and Cyclin D1 in the cervical squamous epithelium among E2-injected Wt, HPV E6/E7 KI and WAPL Tg mice at 6 months after OVX. Left panels show IHC analysis of ESR1, MYC, and Cyclin D1. Insets show higher magnification images. The scale bars represent 50 μm. Right panels show the graphs representing the rates of ESR1, MYC, and Cyclin D1 positive cells in the cervical squamous epithelium. The data were obtained from 6 mice for each group, and the representative images are shown. Error bars on the graphs represent S.D. of the mean. Statistical significance was calculated by Student *t* test. *p < 0.05. n.s. no significant difference.

HPV E6/E7 Tg mice develop CIN by estrogen [14], suggesting that the molecules correlated with HPV E6/E7 are affected by estrogen. On the other hand, we have previously revealed that HPV E6/E7 enhances endogenous WAPL [6]. Moreover, in this study, we demonstrated that WAPL Tg mice developed CIN by estrogen without HPV E6/E7 (Fig. 1). Consequently, WAPL and the estrogen receptor may be correlated. In fact, ESR1 plays a critical role in an early stage of cervical carcinogenesis [16–19]. Therefore, we examined the expression of ESR1 in the cervical squamous epithelium of the E2-injected WAPL Tg mice at 6 months after OVX. Interestingly, the rate of the ESR1 positive cells in cervical squamous epithelium of the OVX-WAPL Tg mice was significantly lower than that in Wt mice (Fig. 2). Furthermore, we examined the expression of MYC, and Cyclin D1, which is regulated by ESR1 [34]. The rate of the MYC and Cyclin D1 positive cells in cervical squamous epithelium of the OVX-WAPL Tg mice was significantly higher than that in Wt mice (Fig. 2).

### Detection of the molecule affecting ESR1 by the high-level expression of WAPL

**Fig. 3:**
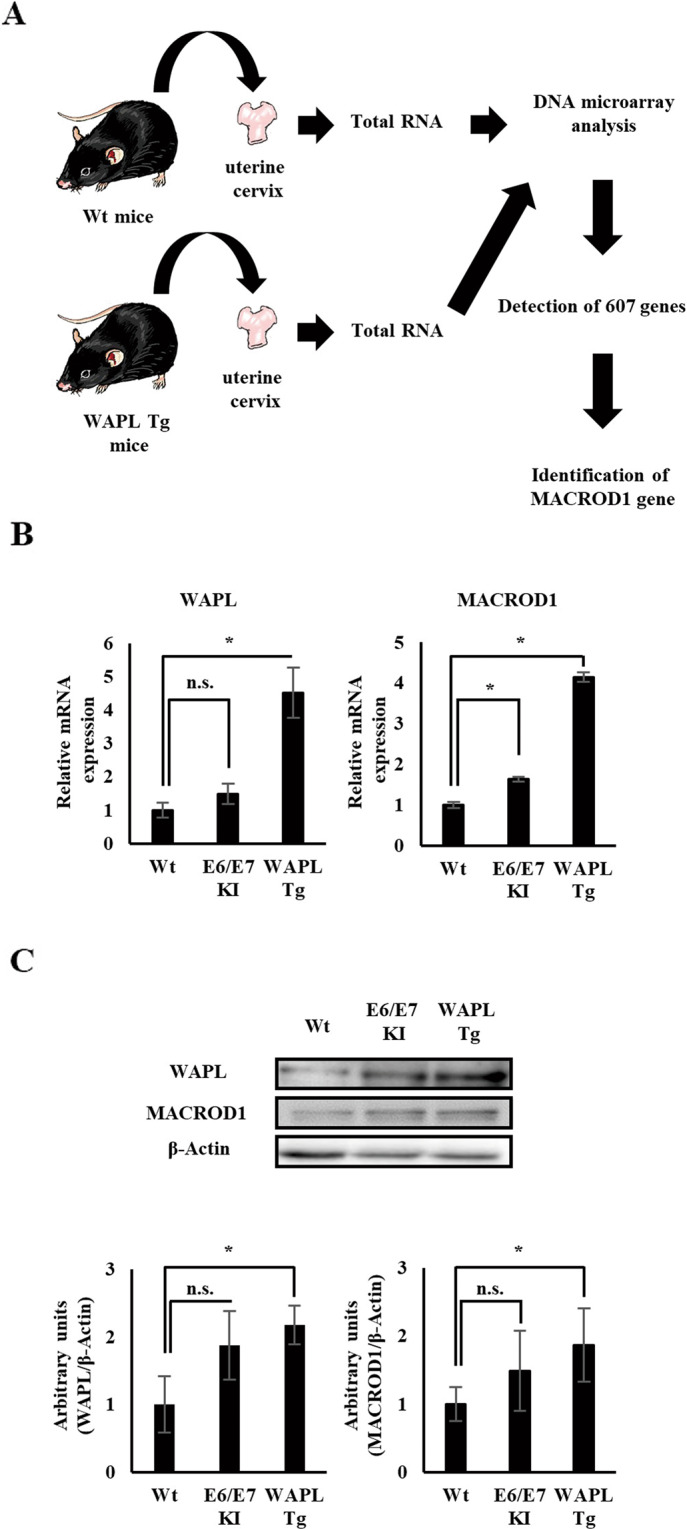
Identification of an ESR1-related factor MACROD1 in the uterine cervix of 6-month-old WAPL Tg mice. **A** Schematic procedure of DNA microarray analysis to obtain the genes that are differentially expressed twofold or more higher in the uterine cervix of WAPL Tg mice relative to Wt mice. **B** Comparison of WAPL and MACROD1 mRNA expression in the uterine cervix among 6-month-old Wt, HPV E6/E7 KI, and WAPL Tg mice by quantitative real-time PCR. **C** Comparison of WAPL and MACROD1 protein expression in the uterine cervix among 6-month-old Wt, HPV E6/E7 KI, and WAPL Tg mice by western blot analysis. **B**, **C** Data were obtained from six mice for each group. Error bars on the graphs represent SD of the mean. Statistical significance was calculated by Student *t* test. *p < 0.05. n.s. no significant difference.

In general, ESR1 expression causes induction of MYC and Cyclin D1. In the above results, however, the expression of MYC and Cyclin D1 in WAPL Tg mice was higher than that of Wt mice, although the ESR1 expression in WAPL Tg mice was significant lower (Fig. 2). Thus, we considered that some kind of molecule enhanced the sensitivity of ESR1 to estrogen by the high-level expression of WAPL. Therefore, to identify such molecules, we performed DNA microarray analysis of gene expression in the uterine cervix of WAPL Tg mice and Wt mice (Fig. 3A). As a result, 607 molecules were highly expressed in WAPL Tg mice compared to Wt mice (Supplemental Table S1). Among them, four factors, IGF1R, IRS2, FOXO4, and MACROD1, are associated with ESR1. Moreover, we focused on MACROD1, which interacts with ESR1 and regulates the expression of MYC and Cyclin D1 [24, 35]. Then, to confirm MACROD1 expression in the uterine cervix of WAPL Tg mice, we performed RT-PCR and western blot analysis. As a result, MACROD1 was significantly higher in WAPL Tg mice than Wt mice (Fig. 3B, C).

### MACROD1 expression is correlated with WAPL in human cervical cancer cells

**Fig. 4:**
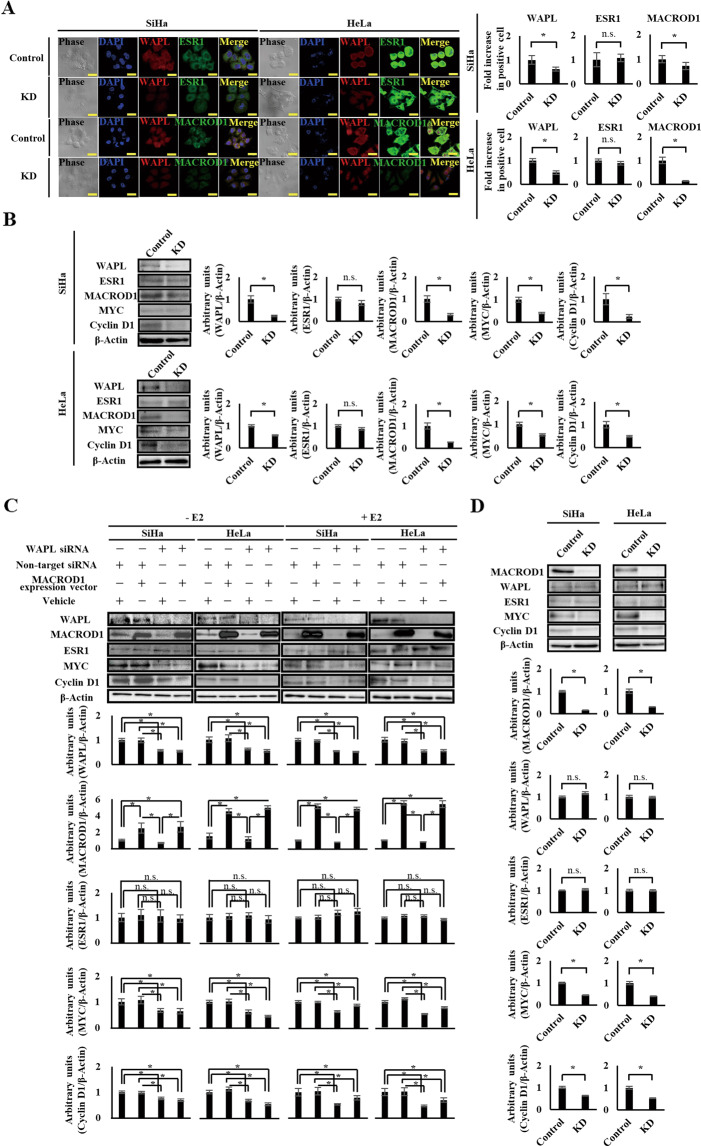
Correlation between WAPL and MACROD1 in human cervical cancer cells, SiHa and HeLa. **A** Immunofluorescence analysis of WAPL, ESR1, and MACROD1 in WAPL knockdown (KD) and non-targeted knockdown (control) cells. Left panel shows representative images of Immunofluorescence analysis from five samples three independent experiments. The scale bars represent 20 μm. Right panels show the graphs representing the ratios of WAPL, ESR1, and MACROD1 positive cells between WAPL KD and control cells. **B** Western blot analysis of WAPL, ESR1, MACROD1, MYC, and Cyclin D1 proteins in WAPL KD and control cells. Left panels show representative images of western blot analysis from six samples in each group and three independent experiments. Right panels show the graphs representing the intensities of signals of western blot analysis. **C** Effect of MACROD1 overexpression on MYC and cyclin D1 expression in WAPL KD cells with and without E2. WAPL siRNA, non-target siRNA, MACROD1 expression vector, and/or vehicle were transfected into SiHa and HeLa cells as indicated above the blot panels. One day after MACROD1 transfection, the cells were treated with or without 100 nM of E2. Then, 2 days after transfection, cells were harvested and subjected to western blot analysis. Upper panels show representative images of western blot analysis from six samples in each group and three independent experiments. Lower panels show graphs representing intensities of signals of western blot analysis. **D** Effect of MACROD1 KD on the expression of WAPL, ESR1, MYC, and Cyclin D1 proteins in cervical cancer cells. Upper panels show representative images of western blot analysis from six samples in each group and three independent experiments. Lower panels show graphs representing intensities of signals of western blot analysis. Error bars on the graphs represent SD of the mean. Statistical significance was calculated by Student *t* test. *p < 0.05, n.s. no significant difference.

MACROD1 is a co-activator of ESR1, and responds to estrogen [24]. However, there are no reports indicating that MACROD1 is associated with the development of cervical cancer. Therefore, to investigate the correlation between MACROD1 and WAPL, we examined the effects of WAPL KD on MACROD1 in a human cervical SCC cell line SiHa and a human cervical adenocarcinoma cell line HeLa. As a result, ESR1 expression showed no significant difference between the WAPL KD cells and non-target KD cervical cancer cells (control cells), but the expression of MACROD1 in the WAPL KD cells was significantly decreased as compared to the control cells (Fig. 4A, B). In addition, we observed a decreased expression of MYC and Cyclin D1 in WAPL KD cells (Fig. 4A, B). These results suggested a correlation between WAPL and MACROD1. Therefore, to further study the correlation WAPL and MACROD1, we overexpressed MACROD1 in WAPL KD-SiHa and -HeLa cells and examined the recovery of expression of MYC or Cyclin D1 with and without E2. The expression of MYC and Cyclin D1 was not induced by MACROD1 overexpression in WAPL KD cells without E2, but was induced with E2 (Fig. 4C). Furthermore, we examined the effects of MACROD1 KD on SiHa and HeLa cells. Although WAPL and ESR1 expression showed no significant difference between the MACROD1 KD cells and control cells, the expression of MYC and Cyclin D1 in the MACROD1 KD cells was significantly decreased as compared to the control cells (Fig. 4D). These results suggested that MACROD1 is regulated by WAPL, and affects the activity of ESR1 downstream molecules such as MYC and Cyclin D1 in human cervical cancer cells.

### WAPL increases ESR1 sensitivity to estrogen by activating MACROD1 in human cervical cancer cells

**Fig. 5:**
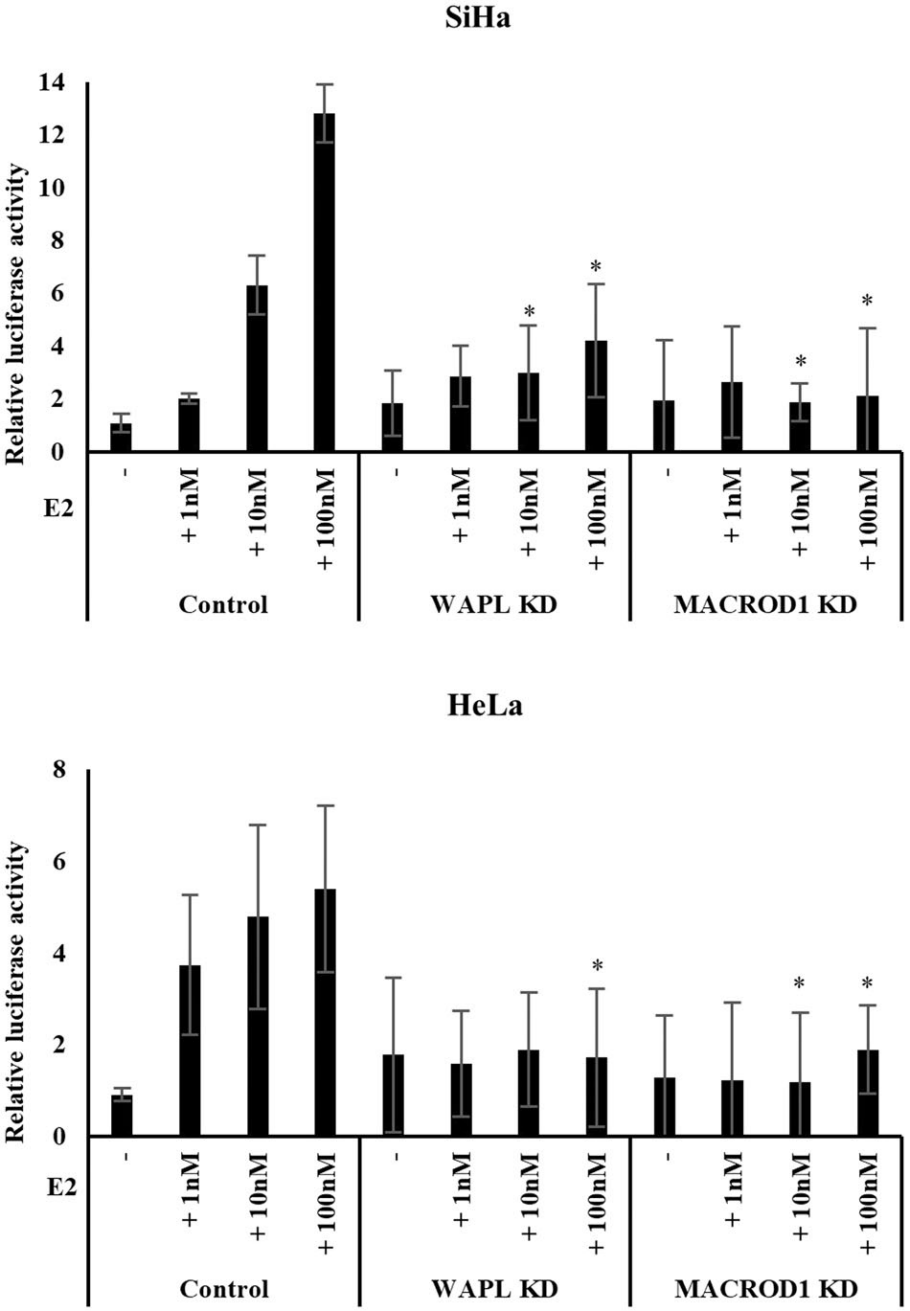
Analysis of ESR1 sensitivity to estrogen in WAPL and MACROD1 KD-SiHa and HeLa cells by luciferase reporter assay. Control is non-targeted knockdown in the cells. Error bars on the graphs represent SD of the mean from six samples in each group and three independent experiments. Statistical significance was calculated by Student *t*-test. *p < 0.05.

As shown in Fig. 2, we found high-level expression of MYC and Cyclin D1 in the cervical squamous epithelium of WAPL Tg mice even though ESR1 exhibited low-level expression. Moreover, MACROD1 showed high-level expression in WAPL Tg mice (Fig. 3B). Furthermore, the expression of MYC, Cyclin D1, and MACROD1 was decreased in cervical cancer cells by WAPL KD although there was no significant difference of ESR1 expression level between WAPL KD and control cells (Fig. 4A–C). From these results, we speculated that WAPL may increase ESR1 sensitivity to estrogen by activating MACROD1. Then, we performed luciferase-based E2-ESR1 reporter assay using WAPL KD and MACROD1 KD human cervical cancer cells. The luciferase activity in E2-treated WAPL KD cells was significantly lower than that in control cells (Fig. 5). Surprisingly, the luciferase activity in E2-treated MACROD1 KD cells was also significantly lower than that in control cells (Fig. 5). These results suggested that WAPL increases ESR1 sensitivity to estrogen by activating MACROD1 in human cervical cancer cells.

### MACROD1 expression shows a similar expression pattern to WAPL in human cervical lesions

**Fig. 6:**
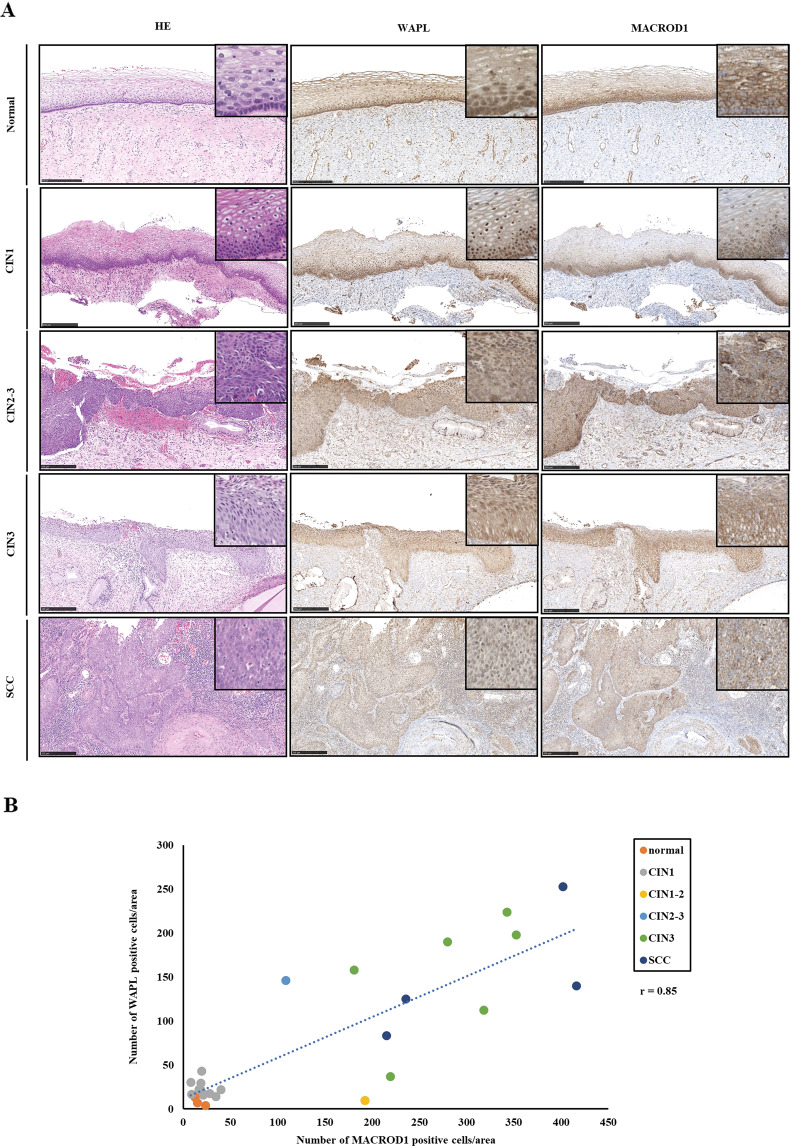
WAPL and MACROD1 expression patterns in human cervical squamous epithelial lesions. **A** HE stain and IHC analysis of WAPL and MACROD1 in Normal, CIN1, CIN2-3, CIN3, and SCC. The scale bars represent 250 μm. **B** Liner correlation between WAPL and MACROD1 positive cells in IHC analysis. Straight lines are fitted to the data by linear regression with Pearson’s correlation coefficients *r* = 0.85, *p* < 0.01. The data were obtained from three patients with normal, 12 patients with CIN1, 1 patient with CIN1-2, 1 patient with CIN2-3, 6 patients with CIN3, and 4 patients with SCC for each group.

In the present study, we showed that WAPL induced MACROD1 expression in the experiments using animal models and human cultured cells. Next, we examined the expression patterns of MACROD1 and WAPL in human cervical lesions by immunostaining. WAPL and MACROD1 showed similar expression patterns in CIN1, CIN2-3, and SCC (Fig. 6A). Furthermore, the Pearson correlation coefficient indicated that MACROD1 expression was positively correlated with WAPL expression in human cervical lesions (Fig. 6B).

## Discussion

Persistent infection with HPV causes CINs in uterine cervix, leading to advanced cervical cancer. In this case, HPV E6/E7 oncoproteins bind to the tumor suppressors p53 and pRB, respectively, resulting in the degradation of p53 and pRB via ubiquitin. Therefore, the carcinogenesis progresses in the same state as the accumulation of mutations in the p53 and pRB genes [36, 37]. We have previously reported that WAPL is induced by HPV E6/E7, is activated in patients with CIN and advanced cervical cancer [5, 6] and causes chromatin instability [13]. However, the mechanism by which activated WAPL progresses CIN and cervical cancer is not fully understood at in vivo level. In this study, we generated WAPL Tg mice overexpressing WAPL in the uterine cervix. We found that high-level expression of WAPL induced CIN in cervical squamous epithelium. On the other hand, accumulation of DNA damage in HPV E6/E7-expressing cells leads to the cell cycle progression and the formation of anaphase bridges between chromosomes during the anaphase of cell division, leading to chromosomal structural abnormalities [38, 39]. Thus, continuous expression of HPV E6/E7 may lead to the accumulation of chromosomal aberrations and high-level expression of WAPL, which may induce cell transformation in uterine cervix. K14-E6/E7 Tg mice, which highly express E6/E7 under the control of the human keratin 14 promoter, develop invasive cancer [40, 41]. However, in the present study, E6/E7 KI mice developed CIN but did not lead to cervical cancer. Thus, we consider that the difference of the expression level of E6/E7 in cervical squamous epithelial cells between K14-E6/E7 Tg mice and E6/E7 KI mice may have caused a difference in the expression level of WAPL induced by E6/E7. In fact, although our previous study has demonstrated that exogenous expression of E6/E7 induce WAPL [6], WAPL expression level in E6/E7 KI mice was not significantly higher than that in Wt mice. We also found that high-level expression of WAPL in the cervix caused CIN. The symptoms were more severe than those of E6/E7 KI mice. In addition, WAPL expression in WAPL Tg mice was higher than that in E6/E7 KI mice, suggesting that the transformation of cervical squamous epithelial cells is WAPL-dependent. Furthermore, we have revealed that WAPL expression pattern is significantly correlated with the severity of human cervical lesions [5]. The expression pattern was also confirmed in the present study. Thus, WAPL may play a key role in cervical oncogenesis.

**Fig. 7:**
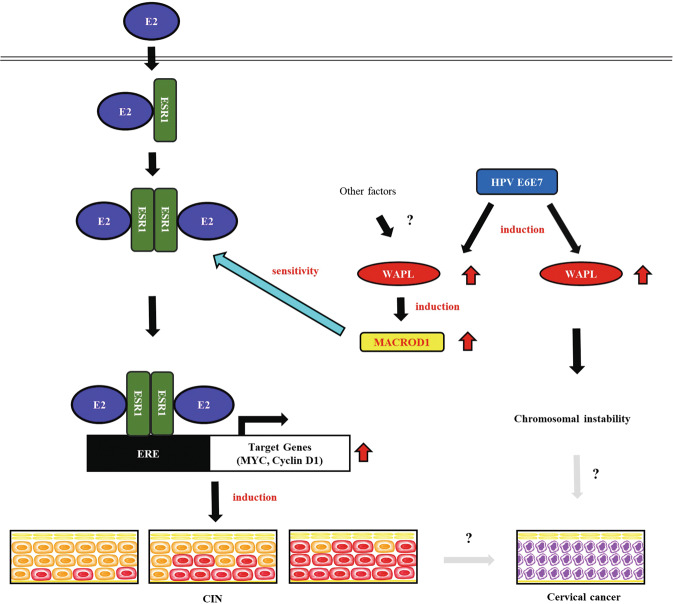
The proposed of mechanism for CIN. In HPV-infected cervical epithelial cells, WAPL plays key roles in the regulation of estrogen sensitivity by activating MACROD1 in early stages of carcinogenesis.

On the other hand, the transcription factor SALL4 is highly activated in various malignant tumors including cervical, liver, lung, gastric, breast, and endometrial cancers [42–47]. SALL4 activates MYC and Cyclin D1 by upregulating the Wnt/β-catenin pathway in cervical cancer cells [42]. In addition, other transcription factors such as NANOG, OCT4, LGR5, and EZH2 promote tumor formation in cervical cancer [48–51]. Thus, these transcription factors may be elaborately associated with WAPL and ESR1. In fact, increased expression of LGR5 was observed in WAPL Tg mice compared to Wt mice by DNA microarray analysis. However, we failed to detect SALL4, NANOG, and OCT4 in human cervical cancer tissues by immunostaining analysis (data not shown). Thus, further experiments are required to elucidate the mechanism. Genes and transcription factors under the control of estrogen receptors are involved in the formation of cervical squamous epithelium [52, 53]. On the other hand, estrogen and its receptors are risk factors that contribute to the development and malignancy of CIN and cervical cancer [14, 15, 17, 54]. In this study, we analyzed the association between estrogen and its receptor in the development of CIN. We found that WAPL increased the estrogen sensitivity of ESR1 by activating MACROD1. Moreover, MACROD1 showed a similar expression pattern to WAPL in various human cervical specimens including normal epithelium, CIN and SCC. On the other hand, overexpression of MACROD1 activates RAC1 and ERK1/2, which are heavily involved in cell proliferation and oncogenic mechanisms [55]. Thus, we concluded that WAPL not only induces chromosomal instability in cervical carcinogenesis, but also plays a key role in the regulation of estrogen sensitivity by activating MACROD1 in early stages of carcinogenesis (Fig. 7). Further analysis of WAPL and MACROD1 may provide a new preventive measure against development of CIN. In conclusion, this study demonstrates that high-level expression of WAPL extends CIN in the cervical squamous epithelium of WAPL Tg mice. Furthermore, WAPL increases ESR1 sensitivity to estrogen by activating MACROD1, and influences the expression of the ESR1 downstream factors MYC and cyclin D1. Thus, we have uncovered a part of the roles of WAPL in the development of CIN and cervical cancer.

## Materials and methods

### MACROD1 expression vector and transfection

The full length of MACROD1 cDNA derived from human non-small cell lung carcinoma cell line H1299 was inserted into the pcDNA 3.1 vector (Thermo Fisher Scientific). The resultant MACROD1 expression vector was transfected into SiHa or HeLa cells using Lipofectamine 3000 reagent (Thermo Fisher Scientific) according to the manufacturer’s recommendation. Subsequently, 100 nM of E2 was added to the cells 1 day after transfection. The cells were collected at 24 h after the addition of E2. The detailed information of the primers to amplify or confirm MACROD1 sequence is described in Supplemental Table S2.

### Animals

All animal care and procedures that were performed in this study conformed to the guidelines for animal experiments of Tokyo Medical University, and they were approved by the Animal Research Committee of Tokyo Medical University (R2-0066). The mice with developmental abnormalities were excluded from experiments. In addition, normal individuals were randomly selected.

### WAPL Tg mice and HPV E6/E7 KI mice

Further details are provided in the Supplementary materials and methods.

### Immunohistochemistry (IHC) analysis

All human cervical specimens were obtained from patients treated at Tokyo Medical University Hospital (Tokyo, Japan) under an approved Institutional Review Board protocol (T2020-0324). The human and murine uterine cervix specimens were fixed with formalin and embedded in paraffin. Sections were stained with Hematoxylin and Eosin or incubated with a primary antibody, and a second antibody. They were visualized using the EnVision DAB kit (Dako A/S, Denmark). Stained images were taken and analyzed by NanoZoomer Digital Pathology software (Hamamatsu Photonics, Japan). The specific information of antibodies is described in Supplemental Table S3. Six mice in each group for scoring of IHC staining, IHC staining cells were counted from five locations randomly selected from the section of the uterine cervix of WAPL transgenic mice and human uterine cervix by ImageJ software. Either nucleus or cytoplasmic signal above background was scored as positive.

### Immunofluorescence microscopy

Three days after transfection of WAPL siRNA, SiHa and HeLa cells were fixed with 1% glutaraldehyde and methanol. After blocking with 1% BSA, the samples were incubated sequentially with primary and secondary antibodies. Then, the samples were analyzed by LSM 710 laser scan microscope (Carl Zeiss, Germany). Fluorescence intensities from images of 5–8 randomly selected microscopic fields of samples were semi-quantitatively analyzed by ZEN lite software (Carl Zeiss). The specific information of antibodies is described in Supplemental Table S3.

### Luciferase reporter assays

25 nM of WAPL siRNA, MACROD1 siRNA, or negative control siRNA was transfected into SiHa and HeLa cells. The next day, pGL4 luciferase reporter vector (Promega, USA) containing estrogen response element-dependent ESR1 was transfected into the cells using the Lipofectamine 3000 reagent (Thermo Fisher). Then, 1, 10, or 100 nM of E2 were added to the cells 2 days after siRNA transfection. The cell extracts were prepared 24 h after the addition of E2, and the luciferase activity was measured using the Dual-Luciferase Reporter Assay System (Promega) according to the manufacturer’s recommendation. All experiments were performed in triplicate.

## Supplementary information

Supplementary Materials and methods

Supplementary Figure 1 legends

Supplemental Figure 1

Supplemental Table S1

Supplemental Table S2

Supplemental Table S3

## Data Availability

Access to the data from our study is available upon reasonable request
