# Supplementary material for: WAPL induces cervical intraepithelial neoplasia modulated with estrogen signaling without HPV E6/E7
Source: Oncogene. 2021 May 4;40(21):3695–706. doi: 10.1038/s41388-021-01787-5 (PMC8154587; doi:10.1038/s41388-021-01787-5)
Supplement: Supplementary file 1 — Supplementary Materials and methods [file 41388_2021_1787_MOESM1_ESM.doc]

**Supplementary Materials and Methods**

**Cell culture** The human cervical cancer cell lines, SiHa and HeLa, were cultured in DMEM (Gibco, USA) with 10% FBS (Sigma-Aldrich, USA) and penicillin-streptomycin (Gibco). SiHa and HeLa were obtained from the American Type Culture Collection (ATCC). All cell lines were examined and authenticated by short tandem repeat profiling. All cell lines were Mycoplasma negative. The mouse ES cells were cultured for generation of HPV E6/E7 KI mice as described previously [1]. **siRNA transfection** SiHa or HeLa cells were transfected with 25 nM of WAPL siRNA, MACROD1 siRNA, or negative control siRNA using Lipofectamine RNAiMAX transfection reagent (Thermo Fisher Scientific, USA) according to the manufacturer’s recommendation. The sense strand sequences of WAPL and negative control siRNAs are 5'-GGAGAUAAAUCAAAGGAAATT-3' and 5'-UACUAUUCGACACGCGAAGTT-3', respectively. MACROD1 siRNA was purchased (EHU121551; Sigma-Aldrich).

**WAPL expression vector**

The full length of WAPL cDNA derived from mouse testis was inserted into the pCXN2 vector [2]. The detailed information of the primers to amplify or confirm WAPL sequence is described in Supplemental Table S2.

**Construction of the HPV E6/E7 knock-in (KI) targeting vector**

HPV E6/E7 cDNA was amplified by PCR using the pDONR201-16E6E7 vector　[3] as a template. Then, to insert into genomic locus of the progesterone receptor, a 2.8-kb short arm was added to upstream of the HPV E6/E7 cDNA, and a 10.0-kb long arm, Poly A tail (PA), Neomycin resistance gene (NEO) and the diphtheria toxin fragment A gene (DT-A) were added to the downstream of the HPV E6/E7 cDNA by the Red/ET recombineering system [1]. The detailed information of the primers used in these methods is described in Supplemental Table S2.

**Validation of homologous recombination by PCR and Southern blot analysis**

To detect the 4.0-kb PCR fragment corresponding to HPV E6/E7 in ES cell clones and the F1 mice derived from the chimeric mice, PCR-based screening was performed using the external ES_E6E7_F1 primer and the internal ES_E6E7_R1 primer. Moreover, the PCR-positive ES cell clones and F1 mice were subjected to Southern blot analysis using the 5' external probe to confirm the integration of the 5'-upstream region of the target sequence and the Neo internal probe to confirm the insertion of Neo. After complete digestion with *Kpn*I, the genomic DNA (10 μg) was separated by electrophoresis on a 0.8% agarose gel and blotted onto a nylon membrane (Pall Corporation, New York, United States) before hybridization with the DIG High Prime DNA labeling and detection starter kit II (Roche, Switzerland). Blotting with the 5' external probe yielded a 21.5-kb fragment corresponding to the wild-type allele, and a 10.4-kb fragment corresponding to the mutant allele. Furthermore, blotting with the Neo internal probe yielded a 10.4-kb fragment corresponding to the mutant allele. The 5' external probe was generated from a 342-bp PCR fragment using the Pr_ex5_4_F primer and Pr_ex5_4_R primer. The Neo probe was generated from an 804-bp PCR fragment using the Neo_F1 primer and Neo_R1 primer. The detailed information of the primers is described in Supplemental Table S2.

**RNA isolation and quantitative real-time PCR**

Total RNA was isolated from the transfected cells and the uterine cervix of the 6 and 7-months-female C57BL6/N mice, E6/E7 KI and WAPL Tg using Isogen reagent (Nippon Gene, Japan). First-strand cDNA was synthesized with Quantitect Rev, transcription kit (Qiagen, Germany) from the total RNA, and used for quantitative real-time PCR (TaqMAN assay or SYBR Green assay). TaqMAN assay probes for β-Actin (Assay ID, Mm01205647_g1; Applied Biosystems, Thermo Fisher Scientific), WAPL (Assay ID, Mm01208743_m1; Applied Biosystems) and MACROD1 (Assay ID, Mm00506589_m1; Applied Biosystems) were used. For SYBR Green assay, the primers of E6_cybr_F1 (5'-ATTAGAACAGCAATACAACAA-3'), E6_cybr_R1 (5'-GCAACAAGACATACATCG-3'), E7_cybr_F1 (5'-ACAGAGCCCATTACAATA-3') and E7_cybr_R1 (5'-CATTAACAGGTCTTCCAA-3') were used for detection of HPV E6 and E7. Quantitative PCRs were run on a 7900HT Fast Real-Time PCR System (Applied Biosystems) and analyzed with SDS v2.4 software (Applied Biosystems). E6, E7, WAPL and MACROD1 mRNA levels were normalized to β-Actin signals.

**Western blot analysis**
 SiHa, HeLa cells and the uterine cervix of Wt, E6/E7 KI and WAPL Tg were lysed in RIPA buffer (Wako Pure Chemical, Japan) with a protease inhibitor cocktail (Sigma-Aldrich). The protein samples were separated by SDS-PAGE and transferred to Immobilon-P Transfer Membranes. The membranes were blocked with 5% skim milk and incubated with a primary antibody at 4°C overnight, and incubated with a secondary antibody for 60 min at room temperature. The protein bands were visualized using ECL select Western Blotting Detection Reagent (GE Healthcare, USA) and ChemiDoc Touch Imaging System (Bio-Rad Laboratories, USA). The intensity of signals was measured by the ImageJ software (National Institute of Health, USA). The specific information of antibodies is described in Supplemental Table S3.

**DNA microarray analysis**
 Total RNA was isolated from the uterine cervix of mice using Isogen reagent (Nippon Gene), and subjected to DNA microarray analysis. DNA microarray analysis was performed by Toray Industries, Inc. Tokyo, Japan. The microarray data have been deposited in the Gene Expression Omnibus (GEO) (http://www.ncbi.nlm.nih.gov/geo/), platform accession number GSE169496.

**Statistical Analysis**

All the statistical analyses in the study were performed using Student’s t-tests. All the experiments were repeated at least for 3 independent times and data were expressed as means ± standard deviation (S.D.). p < 0.05 was considered as a significant difference.

**References**

1. Kumagai K, Takanashi M, Ohno SI, Kuroda M, Sudo K. An improved Red/ET recombineering system and mouse ES cells culture conditions for the generation of targeted mutant mice. Exp Anim. 2017;66:125-136.

2. Niwa H, Yamamura K, Miyazaki J. Efficient selection for high-expression transfectants with a novel eukaryotic vector. Gene. 1991;108:193-199.

3. Teng Z, Yoshida T, Okabe M, Toda A, Higuchi O, Nogami M, et al. Establishment of immortalized human amniotic mesenchymal stem cells. Cell Transplant. 2013;22:267-278.
