# Supplementary material for: WAPL induces cervical intraepithelial neoplasia modulated with estrogen signaling without HPV E6/E7
Source: Oncogene. 2021 May 4;40(21):3695–706. doi: 10.1038/s41388-021-01787-5 (PMC8154587; doi:10.1038/s41388-021-01787-5)
Supplement: Supplementary file 2 — Supplementary Figure 1 legends [file 41388_2021_1787_MOESM2_ESM.doc]

**Supplementary Figures legend**

**Supplementary Fig. 1.**
Generation of WAPL Tg and HPV E6/E7 KI mice. **A** WAPL expression vector for generation of WAPL Tg mice. A 3.6-kb WAPL cDNA inserted into the *Xho*I site downstream of the CAG promoter of the pCXN2 vector. **B** Comparison of WAPL mRNA expression in the uterine cervix between Wild type (Wt) mice and WAPL Tg mice by quantitative real-time PCR. **C** Comparison of WAPL protein expression in the uterine cervix between Wt mice and WAPL Tg mice by western blot analysis. **D** Targeting strategy. Schematic representation of the HPV E6/E7 cDNA inserted into genomic locus of progesterone receptor for generation of HPV E6/E7 KI mice. Targeting vector was designed that HPV E6/E7 cDNA and neomycin-resistant gene (Neo) were inserted in exon 1. Neo was for positive selection. For negative selection, diphtheria toxin fragment A gene (DT-A) was also inserted in the targeting vector. The 5’ probe and Neo probe used for Southern blot analysis are also shown. **E** Southern blot analysis of F1 mice derived from HPV E6/E7 KI chimeric mice. Genomic DNA was digested with *Kpn*I and hybridized with the 5’ probe and Neo probe. Lanes: +/+, wild type; KI/+, heterozygote. **F** Quantitative real-time PCR analysis of E6 and E7 expression in F1 mice derived from HPV E6/E7 KI chimeric mice. The images of western blot analysis in **C** and Southern blot analysis in **E** are representative of three independent experiments. The data in **B**, **C** and **F** were obtained from 6 mice for each group. Error bars on the graphs represent standard deviation (S.D.) of the mean. Statistical significance was calculated by Student t-test. **p* < 0.05. The experimental animals used were 6 months old for each experiment.
