# Supplementary figures and images for: WAPL induces cervical intraepithelial neoplasia modulated with estrogen signaling without HPV E6/E7
Source: Oncogene. 2021 May 4;40(21):3695–706. doi: 10.1038/s41388-021-01787-5 (PMC8154587; doi:10.1038/s41388-021-01787-5)

Supplementary Fig. 1

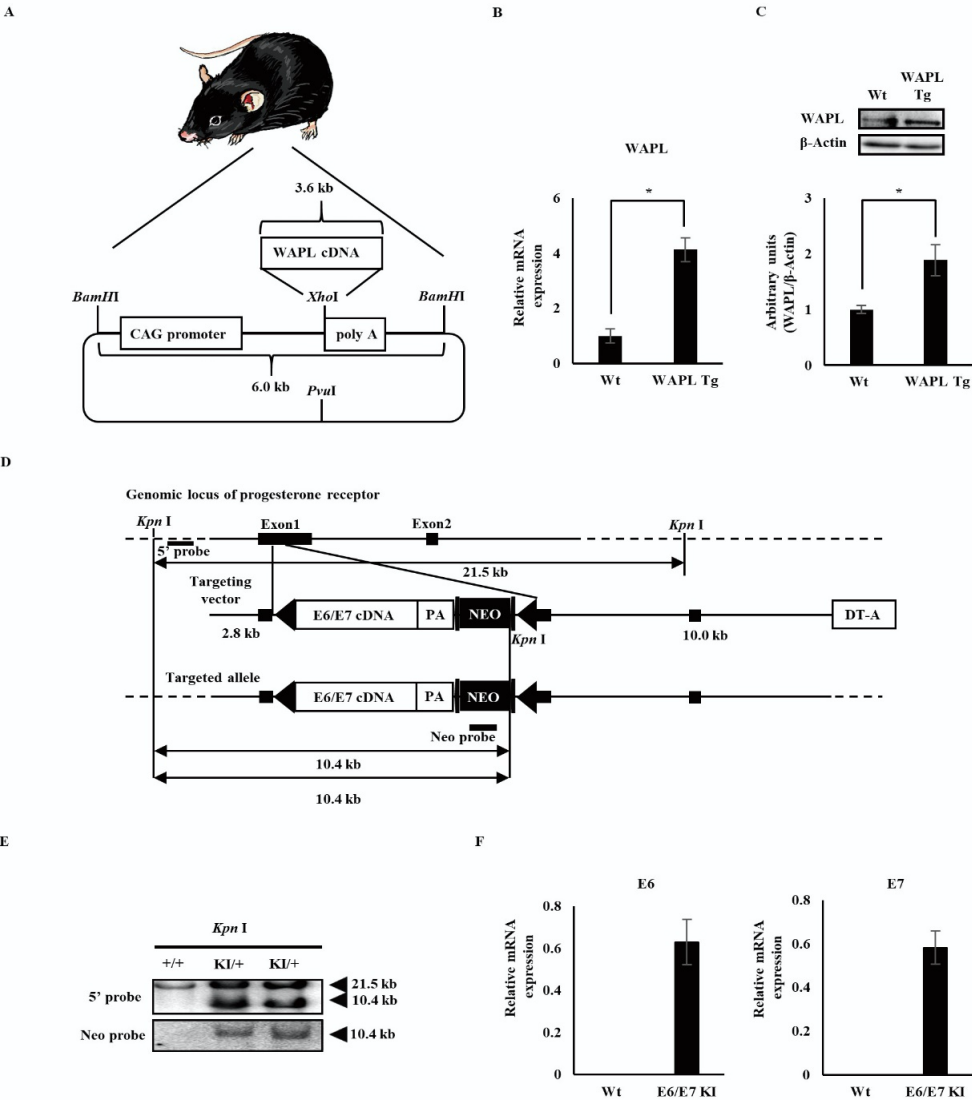

Supplement: Supplementary file 3 — Supplemental Figure 1 [file 41388_2021_1787_MOESM3_ESM.pdf]
