# Supplementary material for: WAPL induces cervical intraepithelial neoplasia modulated with estrogen signaling without HPV E6/E7
Source: Oncogene. 2021 May 4;40(21):3695–706. doi: 10.1038/s41388-021-01787-5 (PMC8154587; doi:10.1038/s41388-021-01787-5)
Supplement: Supplementary file 4 — Supplemental Table S1 [file 41388_2021_1787_MOESM4_ESM.pdf]

Supplemental Table S1

**No.**	1	2	3	4	5	6	7	8	9	10	11	12	13	14	15	16	17	18	19	20
**Symbol**	Cxcl2	S100a9	AA467197	S100a8	Saa3	Cxcl5	Slc26a4	Krt20	Sprr2e	Lgr5	Krt4	Tmc5	Irg1	Il1a	Slc6a14	Cxcl3	Cpm	Cfd	Mup2	Aldh1a3
**No.**	21	22	23	24	25	26	27	28	29	30	31	32	33	34	35	36	37	38	39	40
**Symbol**	Prss22	Sprr2d	Mirt1	Cldn8	Pglyrp1	Duoxa2	Cxcr4	Ccr1	Cyp2e1	Sprr2i	Kcnj15	Ces2c	Rnase1	Zc3h12a	Vnn1	Sp5	Klk1b7-ps	Pcdh19	Bmf	Mefv
**No.**	41	42	43	44	45	46	47	48	49	50	51	52	53	54	55	56	57	58	59	60
**Symbol**	Ces1f	Plet1os	Ces1h	Dio1	Prss27	Cd274	Asprv1	Mup19	Slc36a2	Eda2r	Rnase2b	Mlana	Ihh	Duox2	Slc22a3	Adipoq	St6galnac1	Gabrp	Pglyrp4	Muc20
**No.**	61	62	63	64	65	66	67	68	69	70	71	72	73	74	75	76	77	78	79	80
**Symbol**	Olfml1	Slc34a2	Snx31	Clec4d	Chodl	Fscn2	Plat	Clca3a1	Gpnmb	Hmgcs2	Tlr5	C1qtnf3	Car3	Il1rn	Rad9b	Ifnz	Slc25a27	Clec4e	Dbx2	Cdkn1c
**No.**	81	82	83	84	85	86	87	88	89	90	91	92	93	94	95	96	97	98	99	100
**Symbol**	Rnasel	Sbspon	Pamr1	Fbxw4	Rnase2a	Ypel1	Slc5a8	G0s2	Angptl2	Sycp2	Mybpc1	Cd1d1	Foxq1	Acsm1	Steap4	Mmp15	Lilr4b	Rab15	Noxo1	Gulo
**No.**	101	102	103	104	105	106	107	108	109	110	111	112	113	114	115	116	117	118	119	120
**Symbol**	Psca	Acrbp	Clca4a	Emp1	Anxa1	Sprr2f	Cdh6	Tnfaip2	Art4	Lgr4	Ptgfr	Ptgs2	Olfr338	Fcmr	B3galt2	Muc4	Krt23	Clic6	Klhl41	Ly6e
**No.**	121	122	123	124	125	126	127	128	129	130	131	132	133	134	135	136	137	138	139	140
**Symbol**	Crh	Sell	Abcd2	Dhx57	Mgat4a	Ly6i	Slc46a2	Pik3ip1	Cmya5	Kctd14	Gsto1	Fbxo32	Slc13a2	Defb1	Usp53	Pde5a	Mab21l3	Ccrl2	N4bp2l1	Spink8
**No.**	141	142	143	144	145	146	147	148	149	150	151	152	153	154	155	156	157	158	159	160
**Symbol**	Tril	Kmt2a	Rspo3	Wfdc8	Tnfrsf19	Il1rap	Nfkbiz	Klf5	Zfp945	Adam8	Malt1	Slc15a1	Ear6	Aldh3a1	Oasl1	Tmprss11g	Nfat5	Deptor	Acpp	Sorl1
**No.**	161	162	163	164	165	166	167	168	169	170	171	172	173	174	175	176	177	178	179	180
**Symbol**	Stra6l	Gsto2	Egln3	Lbp	Rasgef1b	H19	Thap6	Mmp9	Eci3	Abca1	Robo1	Pitpnc1	Fam180a	Cadps	Pipox	Ces1d	Shd	Liph	Nlrp10	Hoxa9
**No.**	181	182	183	184	185	186	187	188	189	190	191	192	193	194	195	196	197	198	199	200
**Symbol**	Nox4	Gprc5a	Gltscr1l	Acot1	Mmp14	Inmt	Myoc	Svs4	Vav3	Neto2	Rims1	Lrrn1	Ppbp	Stmnd1	Trp53inp1	Pfkfb4	Fabp7	Npy	Sox1	Hbegf
**No.**	201	202	203	204	205	206	207	208	209	210	211	212	213	214	215	216	217	218	219	220
**Symbol**	Glul	Myl1	Nefm	Hdac11	Zcwpw1	Thrsp	Cyp4a12b	Zfp251	Ell3	Olfr1270	Nfe2l3	Alox12e	Bcl2l11	Aqp4	Ceacam1	Ptgds	Slfn3	Irx5	Tnf	Cyp3a13
**No.**	221	222	223	224	225	226	227	228	229	230	231	232	233	234	235	236	237	238	239	240
**Symbol**	Fmo2	Fgd3	Lpar3	Lepr	Tmem26	Foxo4	Meis2	Scnn1b	Ear2	Ldhb	Adcy6	Car12	Smox	Irs2	Pot1b	Lpl	Pcdh9	Gpr21	Trub1	Cdo1
**No.**	241	242	243	244	245	246	247	248	249	250	251	252	253	254	255	256	257	258	259	260
**Symbol**	Amy1	Pcmtd2	Nsun3	Pbld2	F2r	Sox7	Uox	Ralgps2	Dok5	Slc5a1	Gpr84	Stom	Cfb	Abtb1	Ltbp2	Abcc4	Zfp462	Fam13a	Csf2rb2	Parp8
**No.**	261	262	263	264	265	266	267	268	269	270	271	272	273	274	275	276	277	278	279	280
**Symbol**	Klk9	Gstm1	Olfr1202	Trim31	Neurl3	Capn6	Fam83f	Mesp1	Caprin2	Rnf157	Fat2	Ltf	Epha7	Slc15a2	Dusp5	Klk4	Galnt15	Galr2	Rbm24	Cyp2r1
**No.**	281	282	283	284	285	286	287	288	289	290	291	292	293	294	295	296	297	298	299	300
**Symbol**	Mylip	Sectm1b	Osr2	Dusp26	Rin2	Tgif1	Pigr	Kcne3	Birc3	Trim30b	Meis2	Calcoco1	Dhdh	Arrdc3	Slc25a51	Fam174a	Olfr1382	Ifit2	Chd7	Klhl24
**No.**	301	302	303	304	305	306	307	308	309	310	311	312	313	314	315	316	317	318	319	320
**Symbol**	Defb14	Spink12	Nsg2	Ctsa	Srd5a2	Olfr566	Zfp786	Btnl9	Ankk1	Hsd3b1	Hp	C2	Sik2	Ranbp17	Ecm1	Jag1	Gpr137b	Inf2	Tmem8b	Lrrc1
**No.**	321	322	323	324	325	326	327	328	329	330	331	332	333	334	335	336	337	338	339	340
**Symbol**	Dnajc28	C1qtnf7	Ucp2	Asb11	Il1f9	Dzank1	Tet1	Moxd1	Mr1	Grm5	Dtx3	Sertad4	Foxo3	Npy1r	Speer6-ps1	Ear14	Stard9	Clec5a	Thsd7a	Dapk1
**No.**	341	342	343	344	345	346	347	348	349	350	351	352	353	354	355	356	357	358	359	360
**Symbol**	Setbp1	Fpr2	Pcdh9	Tbc1d8	Ctrc	Six1	Hoxa2	Sord	Adamdec1	Slc1a3	Wfdc5	Sag	Ear12	Chac1	Lrrc46	Cfap206	Fam167a	Stx16	C5ar1	Rasd2
**No.**	361	362	363	364	365	366	367	368	369	370	371	372	373	374	375	376	377	378	379	380
**Symbol**	Adgrg3	Sema4d	Rwdd2a	Cyp26a1	Lgi2	Birc7	Apol7a	Prkg2	Fam102b	Slc6a15	Npas2	Bbox1	Prdm16	Pck1	Tmem45b	Itgbl1	Steap2	Arc	Gstt3	Macrod1
**No.**	381	382	383	384	385	386	387	388	389	390	391	392	393	394	395	396	397	398	399	400
**Symbol**	Gkn3	Pbld1	Nat8f4	Dpysl3	Cyp1b1	Themis3	Pde4b	Arg1	Col23a1	Arhgef6	Rnf39	Heca	Pigc	Efhb	Tnfrsf21	Hoxd4	Map3k7	Dtx3l	Igf1r	Engase
**No.**	401	402	403	404	405	406	407	408	409	410	411	412	413	414	415	416	417	418	419	420
**Symbol**	Rhebl1	Atg9b	Egr1	Myo1g	Prtn3	Nupr1l	Runx1	Tnfsf13	Nkx6-3	Catsper2	Rftn2	Mtmr10	Meis1	Reep6	Lipk	Txnip	Lhfpl5	Pmaip1	Pappa	Trio
**No.**	421	422	423	424	425	426	427	428	429	430	431	432	433	434	435	436	437	438	439	440
**Symbol**	Klk13	Ces2e	Sat1	Rictor	Eaf2	Lrba	Ift22	Cacnb4	Ank3	Phlda1	Epha2	Selenbp2	Cox6a2	Degs2	Zfp36	Rasl11a	Ccng2	Acsl1	Ccnd1	Trmt10c
**No.**	441	442	443	444	445	446	447	448	449	450	451	452	453	454	455	456	457	458	459	460
**Symbol**	Ifna2	Igfbp3	Btbd16	Efcab5	Ces2b	Adamtsl4	Vmn1r72	Cd300ld	Mxd1	Cartpt	Cd36	Rnf167	Olfr441	Ugt1a6a	Mpped2	Rapgef5	Olfr417	Sprr1a	Rps6ka4	Adora1
**No.**	461	462	463	464	465	466	467	468	469	470	471	472	473	474	475	476	477	478	479	480
**Symbol**	Crybg3	Socs7	Slc22a4	Tbrg3	Lbh	Mgp	Trf	Rnf38	Fam134b	Angptl1	Fcna	Caln1	Nlgn1	Gata3	Ppfibp2	Avpr1a	Hmgb3	Lpin1	Grhl2	Prr13
**No.**	481	482	483	484	485	486	487	488	489	490	491	492	493	494	495	496	497	498	499	500
**Symbol**	Adat1	Tinag	Grina	Dmd	Dpep1	Gda	Hcar2	Ankrd42	Nfatc2ip	Zbtb20	Ces1e	Trib1	Stac	Lmx1b	Ppp4r4	Man2a1	Ppm1l	Dhrs4	Casp4	Tbc1d17
**No.**	501	502	503	504	505	506	507	508	509	510	511	512	513	514	515	516	517	518	519	520
**Symbol**	Agtr1a	Stox1	Pnpla1	Sesn1	Flcn	Rgs7bp	Ttc38	Ifi202b	Cbr2	Npr3	Nxf7	Lef1	Olfr346	Pip	Plek	Trpv6	Apln	Higd1c	Zfp2	Rassf10
**No.**	521	522	523	524	525	526	527	528	529	530	531	532	533	534	535	536	537	538	539	540
**Symbol**	Cxcr4	Arid5b	Edil3	Slc18a2	Pdzk1ip1	Airn	Bach1	Tmc4	Dpp4	Tjp3	Tjp1	Gper1	Apobec3	Vdr	Rarb	Mecom	Notum	Eif4b	Baiap2	Osgin1
**No.**	541	542	543	544	545	546	547	548	549	550	551	552	553	554	555	556	557	558	559	560
**Symbol**	Cox8b	Ank2	Aqr	Tifa	Xpnpep3	Ivl	Slco1c1	Hnf1b	Zfp799	Cd300lb	Cib3	Zfp672	Gucy1a3	Dram1	Zswim3	Gbx1	Ssxb9	Spock2	Foxa2	Crebrf
**No.**	561	562	563	564	565	566	567	568	569	570	571	572	573	574	575	576	577	578	579	580
**Symbol**	Sprr3	Selenbp1	Zyx	Rad51d	Sntg1	Dhx38	Pcx	Ank3	Prl3c1	Rnf144b	Hilpda	Zfand5	Vgll4	Atg4c	Sowahb	Tex9	Slfn10-ps	Rassf2	Pgpep1l	Pard6b
**No.**	581	582	583	584	585	586	587	588	589	590	591	592	593	594	595	596	597	598	599	600
**Symbol**	Lfng	Gpr119	Upp1	Card10	Wfdc21	Gpr50	Dpyd	Gpt	Zzef1	Prkar2b	Plxdc2	Ccdc73	Mup4	Olfr644	Olfr1252	Gstm3	Cerk	Rab17	Cd8a	Itgb6
**No.**	601	602	603	604	605	606	607													
**Symbol**	Prss28	Nrg4	Smtnl1	Tmigd1	Adig	Smad3	Lrif1													
