# Supplementary material for: WAPL induces cervical intraepithelial neoplasia modulated with estrogen signaling without HPV E6/E7
Source: Oncogene. 2021 May 4;40(21):3695–706. doi: 10.1038/s41388-021-01787-5 (PMC8154587; doi:10.1038/s41388-021-01787-5)
Supplement: Supplementary file 6 — Supplemental Table S3 [file 41388_2021_1787_MOESM6_ESM.pdf]

Supplemental Table S3

Information of antibody in this study

| Experiments | Antibody | Target protein | Information |
|---|---|---|---|
| Western blot analysis | Primary antibody | WAPL | anti-WAPL antibody (1/1,000 dilution, (Cat.No.16370-1-AP, proteintech Japan, Inc. Tokyo, Japan) |
| | | beta-Actin | anti-beta-Actin antibody (1/1,000 dilution, Cat.No.M177-3, MBL, Nagoya, Japan) |
| | | ESR1 | anti-ESR1 antibody (1/1,000 dilution, Cat.No.ab32063, Abcam) |
| | | MYC | anti-MYC antibody (1/1,000 dilution, Cat.No.ab32072, Abcam) |
| | | Cyclin D1 | anti-Cyclin D1 antibody (1/1,000 dilution, Cat.No.ab16663, Abcam) |
| | | MACROD1 | anti-LRP16 antibody (1/1,000 dilution, HPA041031, Sigma-Aldrich) |
| | Secondary antibody | - | anti-mouse IgG (1/400,000 dilution, Cat.No.A5278, Sigma-Aldrich) |
| | | | anti-rabbit IgG (1/75,000 dilution, Cat.No.A9169, Sigma-Aldrich) |
| IHC | Primary antibody | KRT14 | anti-KRT14 antibody (1/10,000 dilution, Cat.No.ab181595, Abcam) |
| | | Ki-67 | anti-Ki-67 antibody (1/500 dilution, Cat.No.NB-110-89719, Novus Biologicals, Colorad, United States) |
| | | P16 | anti-P16 antibody (1/2,000 dilution, Cat.No.ab54210, Abcam) |
| | | ESR1 | anti-ESR1 antibody (1/200 dilution, Cat.No.NCL-ER-6F11, Leica Biosystems Inc., Illinois, United States) |
| | | MYC | anti-MYC (1/50 dilution, Cat.No.SC-40, Santa Cruz Biotechnology) antibody |
| | | Cyclin D1 | anti-Cyclin D1 antibody (1/100 dilution, Cat.No.ab134175, Abcam) |
| | | WAPL | anti-WAPL antibody (1/1,000 dilution, Cat.No.NBP1-92579, Novusbio) |
| | | MACROD1 | anti-MACROD1 antibody (1/2,000 dilution, Cat.No.HPA041031, Sigma) |
| | Secondary antibody | - | EnVision+ System- HRP Labelled Polymer (Cat.No.K4001, DAKO, Glostrup, Denmark) |
| | | | Swine Anti-Rabbit Immunoglobulins/HRP (Cat.No.P0217, DAKO, Glostrup, Denmark) |
| Immunofluorescence | Primary antibody | WAPL | *anti-WAPL antibody (Cat.No.16370-1-AP, proteintech Japan, Inc. Tokyo, Japan) |
| | | ESR1 | anti-ESR1 antibody (1/200 dilution, Cat.No.ab32063, Abcam) |
| | | MACROD1 | anti-LRP16 antibody (1/200 dilution, Cat.No.ab122688, Abcam) |
| | Secondary antibody | - | Alexa Fluor 488 goat anti-rabbit (1/1,000 dilution, Cat.No.A11034, Invitrogen) |

*10 µg of anti-WAPL antibody (proteintech Japan, Inc. Tokyo, Japan) was labeled by fluorescein labeling kit (DOJINDO MOLECULAR TECHNOLOGIES, Inc. Kumamoto, Japan)
